# Application of Machine Learning to Predict Trajectory of the Center of Pressure (COP) Path of Postural Sway Using a Triaxial Inertial Sensor

**DOI:** 10.1155/2022/9483665

**Published:** 2022-06-22

**Authors:** Kittichai Wantanajittikul, Chakrit Wiboonsuntharangkoon, Busaba Chuatrakoon, Siriphan Kongsawasdi

**Affiliations:** ^1^Department of Radiologic Technology, Faculty of Associated Medical Sciences, Chiang Mai University, Chiang Mai, Thailand; ^2^Research Administration, Academic Services and International Relations Section, Faculty of Engineering, Chiang Mai University, Chiang Mai, Thailand; ^3^Department of Physical Therapy, Faculty of Associated Medical Sciences, Chiang Mai University, Chiang Mai, Thailand

## Abstract

Postural sway indicates controlling stability in response to standing balance perturbations and determines risk of falling. In order to assess balance and postural sway, costly laboratory equipment is required, making it impractical for clinical settings. The study aimed to develop a triaxial inertial sensor and apply machine learning (ML) algorithms for predicting trajectory of the center of pressure (COP) path of postural sway. Fifty-three healthy adults, with a mean age of 46 years, participated. The inertial sensor prototype was investigated for its concurrent validity relative to the COP path length obtained from the force platform measurement. Then, ML was applied to predict the COP path by using sensor-sway metrics as the input. The results of the study revealed that all variables from the sensor prototype demonstrated high concurrent validity against the COP path from the force platform measurement (*ρ* > 0.75; *p* < 0.001). The agreement between sway metrics, derived from the sensor and ML algorithms, illustrated good to excellent agreement (ICC; 0.89–0.95) between COP paths from the sensor metrics, with respect to the force plate measurement. This study demonstrated that the inertial sensor, in comparison to the standard tool, would be an option for balance assessment since it is of low-cost, conveniently portable, and comparable to the accuracy of standard force platform measurement.

## 1. Introduction

Postural control is an essential component in maintaining equilibrium and controlling individual mobility. It is considered a complex integration of multiple systems including motor, higher-level motor systems, and various sensory processes as well as vision, proprioception, and vestibular function [1–3]. The mechanism of postural control plays a key role in maintaining body alignment with respect to gravity, which stabilizes the center of mass (COM) relative to the base of support or limits disturbances in postural stability. Falls are currently a public health issue worldwide, especially among the elderly, who experience adverse effects and their quality of life affected [4]; therefore, the balance and the risk of falls should be evaluated promptly. Qualitative assessments for balance such as the Berg Balance Scale, the Timed-up and Go, and the Functional Reach Test are used commonly in clinical settings [2]. However, these clinical assessments are likely subjective in scoring methods, may show ceiling effects, and are usually insufficient in measuring small degrees of change in the balance performance of the subject [2].

Postural sway reveals stabilizing control in response to perturbations in standing balance and determines the danger of falling. Quantitative measurement of postural sway employs a variety of equipment, including force platforms and stabilometers, to quantify body sway during standing test procedures [5, 6]. A force plate system is recognized as a promising tool for evaluating postural sway [7–9], and the amplitude of the center of pressure (COP) can identify postural stability, which determines the risk of falls in the elderly and individuals with neurological disorders, that is, Parkinson's disease [8–10]. Although the force plate system is the gold standard, it needs costly laboratory equipment and is therefore unsuitable for clinical settings and field research [2, 9, 11].

With recent advances in sensor and data acquisition technology, a wearable inertial sensor was developed as a potential tool for balance, gait, and mobility monitoring. To date, the development of wireless sensors has introduced a new generation of wireless, portable, and inexpensive wearable sensors, with local data storage that enables ambulatory systems for monitoring mobility in daily life [2, 7, 8, 12–15]. Triaxial inertial measurement units (IMUs) are equipped typically with integrated accelerometers, gyroscopes, and magnetometers that measure angular velocity and linear acceleration of body segments. They have been extensively examined and employed to assess postural control affected by aging and neurological diseases, such as Parkinson's and Alzheimer's disease [6, 16–19]. Recently, a novel strategy employing a multijoint network of inertial sensors and unique algorithms has enabled the system to create a wearable biofeedback suit for tracking underwater motion, in order to enhance the patient's effectiveness during aquatic exercise [20]. These wearable sensors are preferable to the laboratory force plate system since they are portable, inexpensive, and can quantify movement in an actual environment [7, 8, 10, 11, 17–19]; however, reliability and validity should be evaluated so that their performance can be compared to a gold standard. The first objective of this study was to evaluate the validity of developing an inertial sensor for assessing postural sway relative to the COP parameter obtained from the standard measure force platform.

Currently, machine learning (ML) is being utilized in clinical research, such as computer-aided diagnosis, medical signal, and image analysis [21]. The use of ML approaches in movement biomechanics is also increasing, and previous research has employed ML-based classifiers to distinguish between falls and other types of daily mobility [13]. Regarding this, the second objective of this study was to apply ML models to predict a COP parameter from inertial sensor metrics and quantify agreement between sway metrics derived from sensor and ML algorithms. If the reliability of the ML approach in predicting the gold standard force-plate derived metrics is proven, it can be utilized as the potential for assessing postural sway without the aforementioned constraints.

## 2. Materials and Methods

### 2.1. Participant

Fifty-three healthy adults aged 20–72 years participated in this study. They comprised 30 males and 23 females with mean age, weight, height, and body mass index of 45.8 ± 6.7 years, 60.68 ± 12.7 kg, 163.0 ± 0.4 cm, and 22.61 ± 3.7 kg/m^2^, respectively. A power analysis based on a prior study of validity [8] indicated that a sample size of 36 would be required for a power of 0.80 and confidence limits within 95%. All of the participants were healthy with no current complaints of weakness, pain, or vestibular dysfunction, and they were not taking any medications that affected postural stability. Elderly participants (aged over 60 years), who were unable to walk independently in the community or had vision impairments or history of falling more than twice in the previous 6 months were excluded from this study. All procedures were approved by the Ethics Committee, Faculty of Associated Medical Sciences, Chiang Mai University, study code AMSEC-63EX-056 (2020), and all of the participants read and completed written informed consent prior to participating.

### 2.2. Protocol and Data Processing

**Figure 1:**
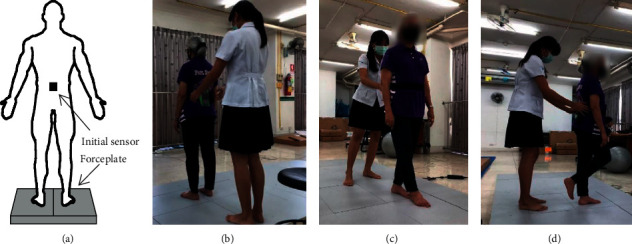
Protocols of the experiment in this study: (a) position of a sensor and force plate; (b)–(d) test conditions during 3 different protocols; double stance standing, standing in tandem, and single-leg stance, respectively.

The participants underwent postural sway assessment by standing on a force platform (PODIUM system, BTS Bioengineering Corp, Italy), with the sensor attached to the lower back at the L5 vertebra near the COM of the human body [17, 22, 23], as shown in Figure 1(a). The force plate was calibrated and configured for accurate COP data prior to performing the tests.

Test conditions comprised the capability of maintaining balance while standing on a firm surface in various progressively challenging positions, double stance standing (Figure 1(b)), standing in tandem (Figure 1(c)) and single-leg stance (Figure 1(d)), in order to challenge balance control (Figure 1). In order to estimate the effects of visual feedback, each task was performed in two tests, one with eyes open and one with eyes closed. Participants performed tests with increasing order of difficulty, starting from a double stance with eyes open (ST-EO), and then with eyes closed (ST-EC); tandem stance with eyes open and closed (TS-EO, TS-EC); and single-leg stance, with eyes open and closed (SL-EO, SL-EC). The tests were performed by standing with bare feet on a platform in order to eliminate variance due to shoe design. The sensor was placed on the lumbar region of the trunk at the L5 vertebra, as recommended [7, 23]. The subjects were asked to maintain an upright standing position and remain as still as possible for each test. Data for each test were captured for 30 seconds, and rest periods between each test lasted 1-2 minutes or until the subjects recovered to a normal or steady condition. All of the participants were instructed to look straight ahead throughout the trial with eyes open. If the participants were unable to perform a trial for 30 seconds, the base of support was reduced to a minimum of 15 seconds. Examples of the protocols are shown in Figure 1.

**Figure 2:**
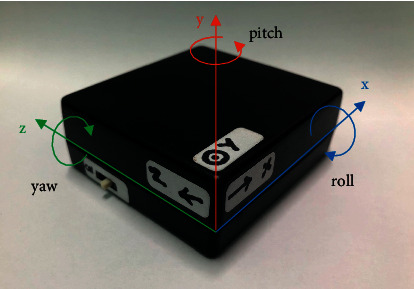
Developed prototype of the triaxial inertial sensor.

**Figure 3:**
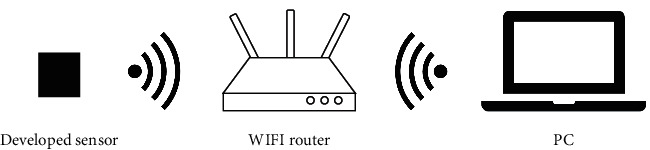
Simple schematic of the connection for data transmission over WIFI between a sensor and a PC.

The developed triaxial inertial sensor (Figure 2) was assembled from 3 parts including (1) a 3-Space Sensor™ Nano, (2) an ESP8266 Node MCU WIFI, and (3) a Lithium polymer battery 3 V 2000 mAh power supply and charging module. Data were acquired with a sampling rate of 10 Hz and transferred to a personal computer (PC) via WIFI as shown in Figure 3.

The COP path (mm) was the sway metric from the force plate and the total length of the COP trajectory in both mediolateral and anteroposterior directions. The three-dimensional geometric-based parameters from the inertial sensor were utilized in this study, as they did not rely on the position of placement. A gyroscope works on all bodies that revolve around an axis, thus developing rotational inertia. It can measure and maintain the orientation and angular velocity of an object in three orthogonal axes; roll, pitch, and yaw. The gyroscope, therefore, is useful for estimating motion direction from various angles, which helps differentiate activities from the angular displacement and exhibits more sensitivity to the sway of COM than an accelerometer (15, 17). The preliminary results of this study supported the hypothesis that all gyroscope-derived parameters show a significant difference (*p* < 0.001) in testing positions, whereas accelerometric results demonstrated no significant difference (*p*=0.170).

The gyroscope outcome measures were the root-mean-square of magnitude (RMS; rad/s), range of magnitude (Range; rad/s), area under the curve of magnitude (AC; rad), and summation of distance (SD; rad/s). Postural sway metrics collected by the inertial sensor were calculated using a custom MATLAB script (Mathworks Inc., Natick, MA).

Data tracking was calculated at the midpoint between 11 and 20 seconds, thus reducing the effect of adaptation and fatigue at the beginning and end of each trial, respectively. The magnitude of each axis was calculated as follows:

(1) $$\begin{array}{c} M_{G}\left(n\right)=\sqrt{G_{x}^{2}\left(n\right)+G_{y}^{2}\left(n\right)+G_{z}^{2}\left(n\right)}, \end{array}$$

where *G*_*x*_(*n*), *G*_*y*_(*n*), and *G*_*y*_(*n*) are the angular velocities in each sample point *n* from *x*, *y*, and *z*-axes, respectively.

The root-mean-square (RMS), range of magnitude (Range), area under the curve (AC), and summation of distance (SD) were calculated using the following equations (equations (2)–(5)):

(2) $$\begin{array}{c} \text{RMS}=\sqrt{\frac{\sum_{n=1}^{N_{s}}M_{G}^{2}\left(n\right)}{N_{s}}}, \end{array}$$

(3) $$\begin{array}{c} \text{Range}=\text{Max}\left(M_{G}\left(n\right)\right)-\text{Min}\left(M_{G}\left(n\right)\right), \end{array}$$

(4) $$\begin{array}{c} \text{AC}=\int_{t=11}^{20}M_{G}\left(t\right)\text{ dt}=\frac{1}{10}\sum_{n=1}^{N_{s}}M_{G}\left(n\right), \end{array}$$

(5) $$\begin{array}{c} \text{SD}=\sum_{n=2}^{N_{s}}\left\|M_{G}\left(n\right)-M_{G}\left(n-1\right)\right\|, \end{array}$$

where *N*_*s*_=90 was the number of sample points of data.

### 2.3. Prediction Models Using ML Algorithms

**Figure 4:**
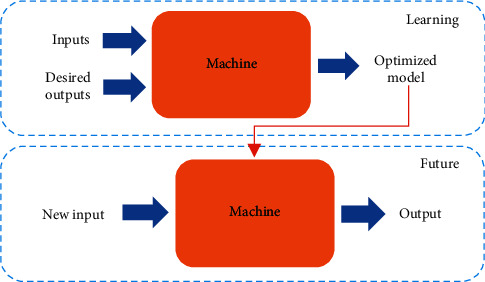
Basic diagram of supervised learning processes.

ML is a subfield of artificial intelligence (AI) that enables software applications to become more accurate at predicting outcomes without explicitly programming them to do so. The primary goal is to allow computers to learn on their own without the need for human intervention. ML employs two basic techniques: supervised learning, which involves training a model on known input and desired output to predict future output, and unsupervised learning, which involves discovering hidden intrinsic structures in input data. As an application in this work required the prediction of suitable future output, supervised learning was applied. The learning or training process began with observations or data (inputs and desired outputs) in order to optimized model and make better decisions in the future, based on new input provided.  Figure 4 shows a basic diagram of supervised learning processes.

Five ML algorithms, that is, least-square boosting (LSBoost), bootstrap aggregation (Bagging), support vector machine (SVM), artificial neural network (ANN), and Gaussian process (GP), were applied in this study for creating prediction models.

LSBoost [24–26] and Bagging [27] are two types of assembled learning methods. LSBoost is a way of combining multiple simple ML models into a single composite one. By combining more and simpler models, the final one becomes a stronger predictor. The term “least-square” refers to the use of algorithms in the Least-square method in order to minimize loss. Simple models (also known as weak learners) were used in this work as decision trees. Bagging is a popular method for reducing variance in a noisy dataset, and in bagging, data in a training set were sampled randomly. After that, weak learners were used to train each sample of data in parallel before combining by using the deterministic average method to obtain a more accurate outcome. Similar to LSBoost, weak learners were decision trees.

The SVM [28, 29] is popular for an ML approach because it produces significant accuracy while requiring low computing costs. The goal of the SVM was to find a hyperplane in N-dimensional space (N: number of input features) that clearly classified the data by maximizing the distance between data points from both classes.

The ANN [30] is a complex adaptive system that may adjust its internal structure based on the data passing through it, which was accomplished by varying the weights of the link. Each weight had a numerical value that controlled the signal between two neurons. All weights in a network were adjusted to improve classification results.

The GP [31] is a nonparametric classification method based on the Bayesian methodology. It assumes the prior distribution of underlying probability densities (normal distribution), which ensures the smoothness of properties. Gaussian or normal distribution was used to fit a given set of training data. The mean of this distribution was then the most likely data characterization. Moreover, a probabilistic approach was used to incorporate the certainty of predictions for good classifications.

The input features of the prediction models and corresponding output were the gyroscope outcome measures and COP path length, respectively. The *k*-fold cross validation method was applied for ML model training [32], which was easy to implement and tended to avoid data selection bias in its procedure. In this work, the ML models were trained with *k* = 5. The optimal parameters of each model were adjusted through experiments.

### 2.4. Statistical Analysis

Identification of concurrent validity of sensor variables to the COP data from the force plate system was performed by Spearman's correlation, a nonparametric measure of correlation, because the dataset violated the assumption of normality. In order to quantify the agreement between sway metrics derived from the sensor and 5 ML algorithms, the intraclass correlation coefficients (ICC_2, 1_), 2-way mixed-effects model, and 95% confidence intervals were conducted [33]. The Bland–Altman graphical plot and scatter plot of correlation were then constructed to determine the agreement between these two quantitative measurements, and bias was defined as the mean of differences between the two measurements [34]. Limits of agreement were calculated as bias ± (1.96 × standard deviation for the difference). Data were analyzed using MATLAB script.

## 3. Results

All selected variables from the sensor prototype, that is, RMS of magnitude, Range, AC, and SD, demonstrated high concurrent validity against the COP path from the force platform measurement, with an excellent correlation coefficient (*r* > 0.75; *p* < 0.001) (Table 1).

From Table 1, output from the inertial sensor could prove its validity against the measures of COP from the force plate by evaluating postural stability during resting stance. Therefore, five ML algorithms were implemented to create prediction models of the COP path.

The parameters in each algorithm were adjusted for the best results, which are known as hyperparameter tuning [35]. In this work, a manual search was used to find suitable parameters for each algorithm, due to the limitation of time and hardware efficacy. The setup of ML algorithms in this study is shown in Table 2.

The ICC_2, 1_ was computed to evaluate agreement between sway metrics derived from the sensor and ML algorithms. All models demonstrated good to excellent agreement between the COP paths from the sensor metrics, with respect to the force plate measurement (ICC; 0.89–0.95).

**Figure 5:**
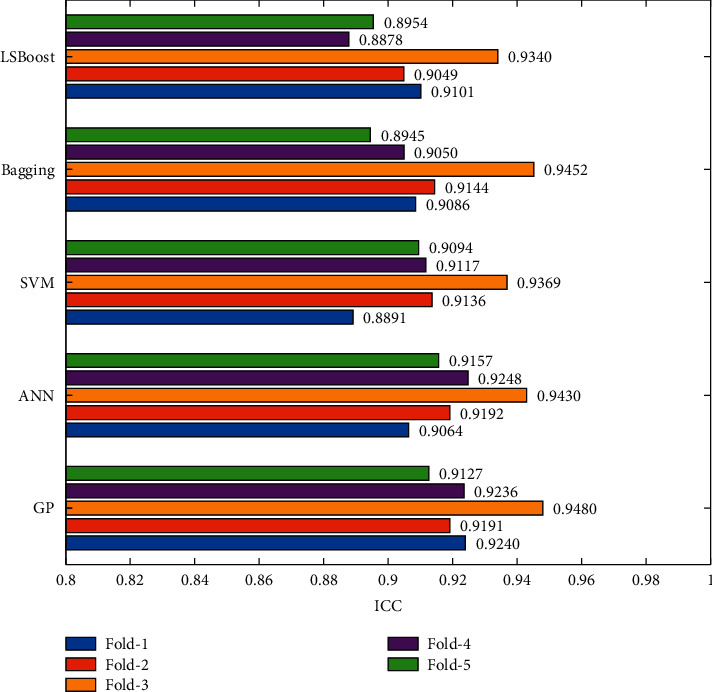
Bar graph showing ICC values from 5 algorithms.

**Figure 6:**
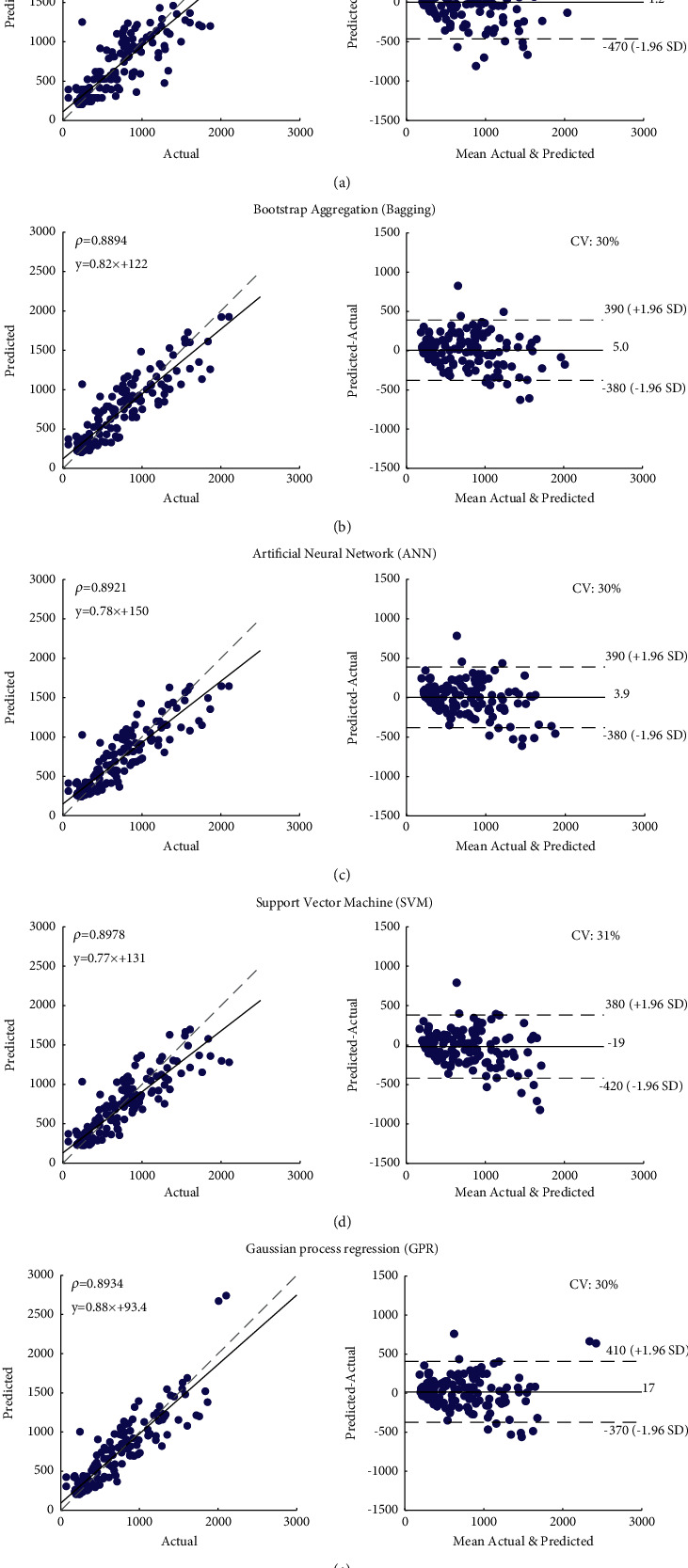
Correlation and Bland–Altman plots evaluated from (a) GB, (b) Bagging, (c) ANN, (d) SVM, and (e) GPR.

The correlation and Bland–Altman plots from the data, which generated the best ICC values of each algorithm (Fold 3 in Figure 5), were evaluated, as shown in Figure 6. The results showed that sensor-gyroscope sway metrics could be used to predict the COP path by good correlation with the COP path from the force plate. Spearman's rho correlation coefficient from all ML algorithms showed excellent correlation (*ρ* > 0.85), and the coefficient of variation (CV) in Bland–Altman plots was approximately 30%.

## 4. Discussion

Since falls among the elderly and other populations at risk continue to be a major source of morbidity and mortality, reliable fall risk assessments should be instituted by healthcare teams for optimal intervention and prevention strategies. The quantitative evaluation of postural control and balance is a clinical implication for all individuals who are at risk of falling. With recent technology, IMUs offer reliable and portable alternatives to force plate-based postural assessment. They are sensitive to early changes in trunk stability by maintaining the COM within the limits of stability. Therefore, capturing trunk sway relative to a base of support during various resting stance positions and sensory conditions can determine impaired postural control and track changes in postural stability during the rehabilitation program. The first objective of this study was to develop a low-cost prototype of an inertial sensor to measure changes in the angular displacement of the triaxial axis while maintaining a certain position and establishing its concurrent validity relative to the COP path length obtained from the force platform measurement. The gyroscope-derived amplitude parameters included in this study were based on the most commonly used parameters for assessing standing balance from the systematic review by Ghislieri in 2019 [8]. All amplitude parameters from the sensor (RMS, Range, AC, and SD) demonstrated significance and excellent correlation (*r* = 0.863–0.892; *p* < 0.001). In accordance with previous studies, the use of an accelerometer with similar parameters (RMS, Range, and SD) found a significant correlation with visual occlusion [17, 36]. Although evaluation of the COM was less common in research settings when compared to force plate-based measures, which record the COP trajectory of the ground reaction force, the point of resultant ground reaction forces using wearable sensors is practical, inexpensive, and easily transportable [29]. The relationship between the COP and COM during body sway in a resting stance could be explained by the motion of an inverted pendulum pivoted at the ankle joint, moving in a sagittal (anteroposterior, A-P) plane. However, with advancing age, the use of hip strategy in a mediolateral (M-L) plane to control stance posture has been reported [17, 30]. A recent review of six studies on static balance in 2021 by Baker demonstrated that the inertial sensor provided moderate to strong evidence of concurrent validity for M-L and A-P sway [37]. Moreover, A-P angular displacement was found to be a significant predictor of falls among elderly adults [32].

The second objective included the above gyroscope sway metrics as input features and applied ML models to predict the COP path output. Several approaches have been reported in the literature, such as the SVM and ANN, which are used widely as classifiers and have shown promising results in problems of pattern recognition in neurological and psychiatry diseases [38]. Nevertheless, the selection of ML approaches is still inconclusive. This study applied 5 common algorithms, that is, LSBoost, Bagging, SVM, ANN, and GP, which were then compared based on their performance in predicting the COP metrics of body sway. The correlation between the actual and predicted values from all algorithms is high (*ρ* > 0.85 and CV%∼30%), as shown in Figure 6. The performance of the model was represented by the slope (*m*) and offset (*c*) values of the linear equation (*y* = *mx* + *c*), in which the slope should be close to 1, and the offset should approach 0. Results from this study showed that the GP algorithm represented the best prediction model since the slope was closest to one with the offset being the smallest (*m* = 0.88, *c* = 93.4). This study demonstrated that the application of ML can predict COP metrics of static standing postural control from a wearable sensor. All 5 algorithms demonstrated similar results, but the GP algorithm performed better than the other models.

A previous study [38] reported the advantage of using more than one ML algorithm to avoid the risk of selection bias. Moreover, the same ML algorithm can generate different results across various experimental settings. Therefore, no comparison was made between ML algorithms in different studies, and only one could be selected. For this reason, five popular ML algorithms were applied and compared in this study. The use of only four parameters as input features of ML from inertial sensors was a limitation of this study. The addition of one or more advanced parameters may improve the prediction performance, for example, those in the frequency domain. Another limitation was that subclassifications of the algorithms were not examined, and hyperparameters were adjusted roughly by the manual search method. For example, no comparison was made between the kernel functions used in the SVM and weak learners in the assembled learning method (LSBoost and Bagging). Only 4-5 values were adjusted for the number of layers and nodes for the ANN and/or learning cycles for LSBoost and Bagging, based on previous reports and the authors' previous research experience. A grid or random search [35] for hyperparameter optimization may improve the performance of each algorithm. However, that would come at a higher cost in terms of resources and time of usage.

In addition to the input features of ML, the type of ML algorithms, and the hyperparameter tuning, the distribution of collected data and the sampling rate also influence the accuracy of COP prediction. As shown in Figure 6, the data collected was dense in the range where the COP trajectory was less than 1,000 since the subjects in this study were healthy adults that had good balance (low COP trajectory). Most of the prediction errors occurred in the COP trajectory greater than 1,000. The lower sampling rate compared to commercial sensors considered an additional limitation of our investigation. It may account for the omission of some data; thus, in the future, we propose to increase the sampling frequency of the developed sensor by modifying its hardware and software.

Although it has recently been demonstrated that using multisensor data fusion has higher recognition accuracy for complex human movements than a single sensor [15], our study aimed to examine the trajectory of sway during quiet stance, which had no movement complexity. Therefore, we positioned a single sensor in the middle of the user's waist, at the L5 vertebra in the lumbar area of the trunk, as evidenced by a previous study that indicated that the central sensors had a higher recognition rate than the left and right ones [23]. However, the development of the system using multiple sensors that could possibly monitor dynamic mobility during tasks such as walking or identify the gait parameters is still interesting. Therefore, our developed multisensors combined with the ML algorithms have been planned for future work.

This study developed a prototype of the triaxial inertial sensor and applied ML approaches to identify postural control during stance and predict the trajectory of COP, which is a resultant ground reaction force. It exhibited the validity of the inertial sensor in relation to laboratory-based measures of postural sway, and the feasibility of using ML models to predict the force plate-derived COP metric from the inertial sensor output. The results of this study offered a promising wearable, the miniature inertial sensor that could be an advantageous alternative to the laboratory-based force platform system for quantitative analysis of static postural sway.

## 5. Conclusion

According to the findings of this investigation, we confirmed the findings of earlier studies [8, 17, 39, 40] in that a triaxial inertial sensor is a viable optional device for assessing postural sway. The study demonstrated the validity of the inertial sensor as well as the plausibility of applying machine learning models to predict the force plate-derived COP metric from inertial sensor output. It should be improved further so that it can be used as a screening tool for individuals who are at risk of falling.

## Figures and Tables

**Table 1:** Spearmen's rho correlation coefficient of inertial sensor variables compared with the COP path derived from a force plate.

Sensor variable	*r*	*p*-value
RMS (rad/s)	0.891	<0.001
Range (rad/s)	0.863	<0.001
AC (rad)	0.881	<0.001
SD (rad/s)	0.892	<0.001

**Table 2:** The parameters of ML algorithms.

ML algorithms	Parameter setting
LSBoost	No. of weak learners: 100 weak learners: decision trees
Bagging	No. of weak learners: 100 Weak learners: decision trees
SVM	Kernel function: rbf Epsilon: tenth of the standard deviation using the interquartile range of the desired outputs
ANN	Network type: feedforward No. of layers and nodes: 2 layers, [3 4] nodes
GP	Kernel function: squared exponentially Sigma: standard deviation of the desired outputs divided by $\sqrt{2}$ Basic function: constant = 1

## Data Availability

The authors have no data other than study results.
